# SWAG: long-term surgical workflow prediction with generative-based anticipation

**DOI:** 10.1007/s11548-025-03452-8

**Published:** 2025-06-26

**Authors:** Maxence Boels, Yang Liu, Prokar Dasgupta, Alejandro Granados, Sebastien Ourselin

**Affiliations:** Surgical and Interventional Engineering, School of Biomedical Engineering and Imaging Sciences, Kings College London, London, USA

**Keywords:** Surgical workflow anticipation, Surgical phase recognition, Surgical workflow generation, Remaining time regression, Cholec80, AutoLaparo21

## Abstract

**Purpose:**

While existing approaches excel at recognising current surgical phases, they provide limited foresight and intraoperative guidance into future procedural steps. Similarly, current anticipation methods are constrained to predicting short-term and single events, neglecting the dense, repetitive, and long sequential nature of surgical workflows. To address these needs and limitations, we propose SWAG (surgical workflow anticipative generation), a framework that combines phase recognition and anticipation using a generative approach.

**Methods:**

This paper investigates two distinct decoding methods-single-pass (SP) and autoregressive (AR)-to generate sequences of future surgical phases at minute intervals over long horizons. We propose a novel embedding approach using class transition probabilities to enhance the accuracy of phase anticipation. Additionally, we propose a generative framework using remaining time regression to classification (R2C). SWAG was evaluated on two publicly available datasets, Cholec80 and AutoLaparo21.

**Results:**

Our single-pass model with class transition probability embeddings (SP*) achieves 32.1% and 41.3% F1 scores over 20 and 30 min on Cholec80 and AutoLaparo21, respectively. Moreover, our approach competes with existing methods on phase remaining time regression, achieving weighted mean absolute errors of 0.32 and 0.48 min for 2- and 3-min horizons.

**Conclusion:**

SWAG demonstrates versatility across generative decoding frameworks and classification and regression tasks to create temporal continuity between surgical workflow recognition and anticipation. Our method provides steps towards intraoperative surgical workflow generation for anticipation. Project: https://maxboels.com/research/swag..

**Supplementary Information:**

The online version contains supplementary material available at 10.1007/s11548-025-03452-8.

## Introduction

Surgical workflow anticipation has the potential to enhance operating room efficiency and patient safety by predicting future surgical events during procedures. Anticipation allows for better preparation, reduces cognitive loads, and improves coordination among surgical teams [1, 2]. While preoperative planning provides an initial framework, it often falls short in addressing the dynamic environment and decision-making required during surgery.

Current approaches predominantly focus on surgical phase recognition, identifying the present phase, step, or action [3–5]. These methods are valuable for postoperative analysis but offer limited assistance in real-time intraoperative decision-making. They cannot anticipate future events to allow for dynamic planning adjustments, which could potentially improve surgical outcomes. Remaining surgery duration (RSD) estimation has been actively researched for over two decades [6–11], demonstrating clinical interest in long-term predictions, i.e. predicting the remaining time until the end of the surgery. Other studies explored surgical workflow anticipation, such as predicting the next phases and instrument occurrences [12, 13]. However, these methods are limited by their requirement to predict a single event per class, disregarding scenarios where multiple future occurrences may exist within a fixed time horizon.

Generative models, such as GPT models [14], address this challenge by generating sequences of tokens that can vary in both length and class frequency. In surgical workflow anticipation, autoregressive (AR) [14] and single-pass (SP) decoding models [15] demonstrate the potential to predict continuous sequences of surgical actions. Unlike conventional remaining time regression approaches, generative models output multiple tokens, extending the observed sequence of events to a plausible future representation of the surgical workflow.

In this work, we present SWAG (Surgical Workflow Anticipative Generation), a generative model designed to unify phase recognition and anticipation in surgical workflows. Our main contributions are as follows:

1. We introduce SWAG, a generative model that combines surgical phase recognition and anticipation, for long-term sequential future prediction of the workflow.
2. We provide an extensive comparison of two generative decoding methods: single-pass and autoregressive—alongside two tasks-classification and regression—to generate continuous sequences of present and future surgical phases.
3. We propose a novel prior knowledge embedding method using future class transition probabilities improving predictive performances.
4. We conduct a comprehensive evaluation on the Cholec80 and AutoLaparo21 datasets, demonstrating SWAG’s capabilities on different surgical procedures.

## Related work

*Surgical phase recognition*: Early research on surgical phase recognition relied on probabilistic graphical models with instrument usage [16]. Later, convolutional neural networks were used to learn spatial representations from surgical video frames, while temporal relations among video frames were captured with recurrent neural networks [17]. Temporal convolutional networks were later able to increase the receptive field [3]. Recent works have successfully used Transformers [18, 19] to capture critical information between frames using short attention windows like in Trans-SVNet [20] and LoViT [4], whereas SKiT [5] proposed an efficient long-term compression approach achieving state-of-the-art performance on online surgical phase recognition. SAHC [21] proposes to model surgical workflows at both frame and segment levels to better capture phase transitions. Limitations between anticipation and batch normalisation for surgical workflow analysis are presented in BNPitfalls [22]. SurgFormer [23] introduces a transformer architecture with hierarchical temporal attention for phase recognition. Although these approaches offer substantial advancements for postoperative video analysis, we focus on intraoperative decision-making by adding anticipative capabilities to the recognition system. Anticipating the surgical workflow over long-time horizons could enhance real-time decision support by forecasting upcoming surgical phases, enabling smoother, more responsive guidance during procedures.

*Surgical workflow anticipation*: Most anticipation approaches in surgery have explored surgical workflow anticipation by predicting the remaining time until the end of surgery [6–11], next instruments [12] or next phases occurrence [13, 24, 25]. Bayesian [12] was proposed to anticipate tool usage which was then used as a baseline in IIA-Net [13] for surgical phase anticipation. IIA-Net [13] was then introduced to leverage instrument interaction for next-phase occurrence regression. Although IIA-Net requires pre-trained models for tool detection and segmentation, we compare our method to this approach and their implementation of Bayesian [12]. Both methods were formulated as a regression problem, for surgical phase anticipation. SUPR-GAN [24] uses LSTMs within an encoder-decoder architecture to predict surgical phases over 15 s. A discriminator is used to train the model, using a generative adversarial network (GAN) approach. In contrast, our SWAG model addresses long-term surgical workflow anticipation, predicting sequences up to 60 min while unifying recognition and anticipation tasks. Zhang et al. [26] propose a graph network with bounding boxes as inputs to predict the occurrence of instruments or phases within 2-, 3-, and 5-min horizons. Later, hypergraph transformer (HGT) was proposed in  [27] to detect and predict action triplets over 4 s. Other methods were also proposed for gesture anticipation as low-level motion planning [28], instrument trajectory prediction [29, 30]. Most methods focus on the next occurrence regression, failing to provide a comprehensive view of the entire surgical workflow, leaving a blind spot for events beyond the first predicted occurrence. Unlike previous works, SWAG uses a generative approach for future phase classification. Our approach addresses some gaps and limitations by predicting sequences of arbitrary length and frequency, and unifying recognition and anticipation tasks.

## Methods

In this section, we present our proposed method for jointly addressing surgical phase recognition and anticipation. Our model, SWAG, is designed to predict the current phase while anticipating the occurrence of future phases over long-time horizons.

### Task formulation

Our primary task is to predict the current and future surgical phases over a horizon of $N = h_N$ minutes, conditioned on observed frames $X_t = \{x_0,\ldots ,x_t\}$, where $x_i \in \mathcal {X}_T$ represents all video frames from the surgical procedure. We define the mapping function as follows:

1 $$\begin{aligned} f_{\theta }: X_{\le t} \mapsto \bigl (y_{t+h_0\cdot 60}, \ldots , y_{t+h_N\cdot 60}\bigr ) \end{aligned}$$

where $X_{\le t}$ denotes the sequence of observed frames up to time $t$. The anticipation sequence $\{h_n\}_{n=1}^{N}$ is a predefined set of time steps, where each $h_n$ corresponds to the number of minutes into the future for which we predict the surgical phase. The sequence is defined as:

2 $$\begin{aligned} h_0 = 0, \quad h_1, h_2, \dots , h_N \in \{1, 2, \dots , N\} \end{aligned}$$

where $h_0$ represents the current phase prediction and $h_N$ defines the maximum anticipation horizon.

For the time regression task, we predict the remaining time until each phase’s next occurrence within a fixed horizon of $[0, N]$ minutes, where $0$ indicates the phase is currently active, and $N$ means it will not occur within the horizon. Our setup follows [12, 13]. We predict phases every 60 s based on our ablations (see Fig. 4 in SM).

**Fig. 1:**
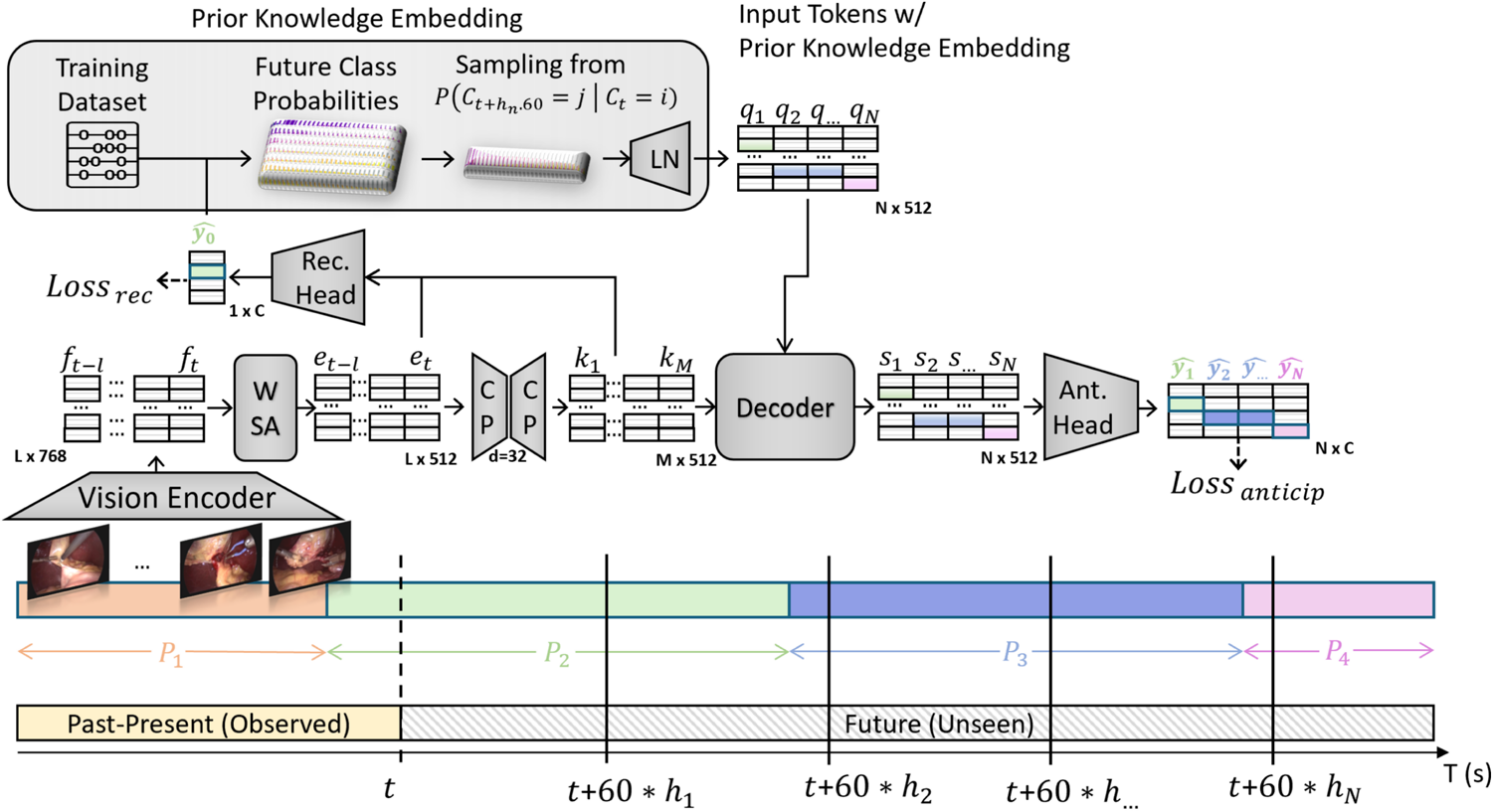
Overview of the SWAG-SP* model architecture. The model processes surgical video data in two main paths: (1) A recognition path with $L=24*60$ frame embeddings and $M=24$ compressed frame representations are encoded into $D=512$-dimensional embeddings through a vision encoder, a windowed self-attention (WSA) encoder and a compression and max-pooling (CP) bottleneck to recognise the current surgical phase with a classification head (loss$_{recognition}$). (2) A generative path samples *N* times from future class probabilities extracted from the training set. These probabilities are combined with temporal position embeddings to form input tokens $Q_t=\{{q_1, q_2, ..., q_N}\}$ and passed to a decoder that conditions the compressed frame embeddings i.e. context tokens $K_t=\{{k_1, k_2,..., k_M}\}$ to predict *N* future surgical phases at 60 seconds intervals (Loss$_{anticipation}$). The timeline shows the model operating on past-present observed frames from phases P$_1$ (orange) and P$_2$ (green) and predicting probabilities $N \times C$ of future phases

### SWAG model architecture

Our proposed model, illustrated in Fig.   1, includes a vision encoder followed by a windowed self-attention encoder (WSA), then a linear Compression and max-pooling block (CP) [5], and finally a future decoder module that can operate either in a single-pass (SP) or an autoregressive (AR) approach. While our model uses classification for the phase recognition task, it can use classification or regression for phase anticipation. This architecture also includes a novel prior knowledge embedding relying on the predicted current class $\hat{y}_{t+h_0\cdot 60}$ and future indices from $h_1$ to $h_N$.

*Vision encoder*: Similar to LoViT, we fine-tune a pre-trained ViT [19] using the AVT [31] approach, which trains on short segments of consecutive frames. This procedure allows the model to incorporate limited temporal context while learning spatial features. At inference, we then extract a single 768-dimensional embedding per frame $f_t$, serving as the initial feature representation.

*Window self-attention (WSA)*: To recognise the observed frame at time *t*, we use the extracted features as input sequence $F_t = {\{f_{t-L}, \ldots , f_t}\}$ with a length of $L=1440$ and $D=768$ dimensions. We then use a sliding window of width $W=20$ to perform self-attention over the extracted image features with no overlap between windows and get an output sequence of the same length and dimensionality, $E_t = {\{e_{t-L}, \ldots , e_t}\}$.

*Compression and pooling (CP)*: We implement two temporal pooling strategies: global key-pooling [5] and interval-pooling, which are used differently in our single-pass and autoregressive decoders. The single-pass method exclusively uses global key-pooling, while the autoregressive approach leverages both methods. Specifically, the recognition branch in both models uses global key-pooling. For global key-pooling, the temporal features $E_t = {e_{t-L}, \ldots , e_t}$ are first projected into a lower-dimensional latent space ($d=32$) as $E'_t$ using a linear layer, then compressed via a cumulative max-pooling approach into *M* tokens $K_t = {k_{1}, \ldots , k_M}$. Following [5], this involves first computing a single pooled representation of the compressed features: $p = \max {\{e'_{t-L}, \ldots , e'_{t-M}\}}$ Then, for each of the most recent *M* frames, we compute a cumulative maximum: $k_m = \max {\{p, e'_{t-M+1}, \ldots , e'_{t-M+m}\}}$ for $m = 1, 2, \ldots , M$ This creates tokens that progressively incorporate more recent information while maintaining context from all previous frames. The autoregressive approach, trained with causal masking on input sequences, requires temporal consistency between the training sequence and the generated outputs tokens during inference. Therefore, we developed *interval-pooling* to aggregate spatial information at regular temporal intervals. Interval-pooling performs max-pooling over consecutive 60-second intervals, with a fixed start time at $t-L$. For each new token, the right-side index extends by 60 seconds, effectively compressing 60 frame embeddings into a single token. This yields *M* context tokens, where *M* corresponds to the sequence length divided by the 60-second interval (e.g. compressing 24 min into 24 tokens: $\frac{1440\text { s}}{60\text {s}}$). This approach ensures a progressive and cumulative aggregation of information which is consistent with the time interval in the generated sequence during inference.

**Fig. 2:**
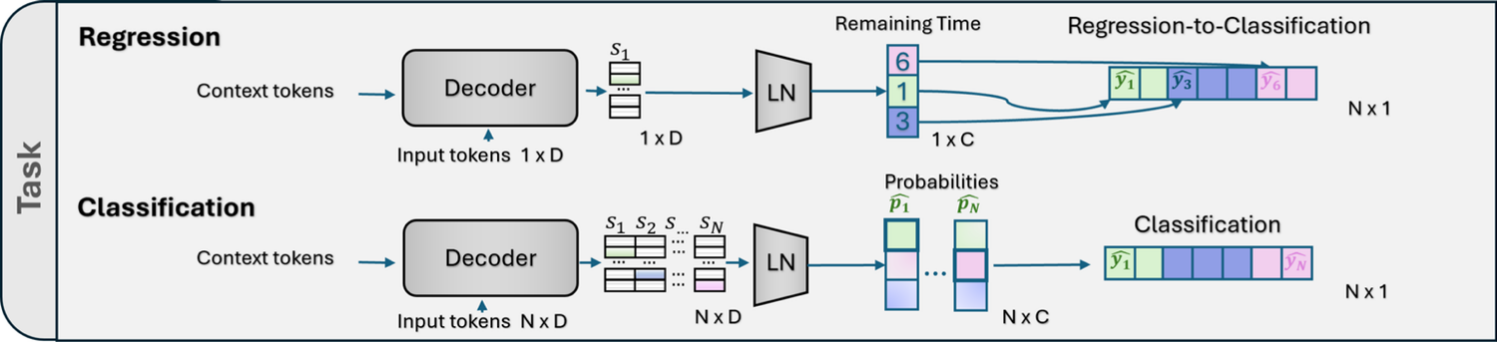
Single-pass regression and classification tasks. The regression task (top) uses a single $1 \times D$ input token to predict the remaining time before each next class occurrence, which can be transformed into a classification task using our regression-to-classification (R2C) method. The direct classification task (bottom) uses *N* input tokens to predict *N* minutes in the future

*Decoder*: Our two decoding approaches (see Fig. 2) can be described as follows:

(1) *SWAG-SP (Single-Pass)*: The vanilla transformer decoder receives *N* input tokens $Q_t=\{{q_1,..., q_N}\}$ and generates *N* output tokens $S_t=\{{s_1,..., s_N}\}$ in a single forward pass, each token represents 60 seconds over *N* minutes. During training, we use a special teacher-forcing approach, using the ground-truth current phase $y_t$ to sample from the correct conditional probability distribution $P(y_{t+h_n\cdot 60} = j \mid y_t = i)$ at future minute index $h_n$, *N* times. The $Q_t$ inputs are initialised with prior knowledge corresponding to conditional future class probabilities extracted from the training set (see Sec. 1.2 in SM) and sinusoidal positional encoding. This generative approach is conditioned using cross-attention between the context tokens $K_t = {\{k_{1}, \ldots , k_M}\}$ and the $Q_t$ inputs to generate all future tokens $S_t$ in a single forward pass, allowing for efficient parallel computing and reducing inference time.

(2) *SWAG-AR (Autoregressive)*: We use the GPT-2 model as our autoregressive decoder [14] which uses causal masking on the features extracted with interval-pooling to predict the next token in the sequence. Teacher forcing is not used as our approach uses the learned frame embeddings from the recognition task rather than embedding ground-truth labels as inputs. During inference, the decoder iteratively uses past predictions as inputs until its generate $N=h_N$ future tokens as its anticipated horizon.

*Recognition Head (Rec. Head)*: We fuse the key-pooled features $K_t$ with the windowed self-attention features $E_t=\{{e_{t-M-1},..., e_t}\}$ using a skip connection [5]. The resulting fused feature vectors are then passed through a classification layer to predict *M* class probabilities $\{\hat{p_1}, \ldots , \hat{p_M}\}$, which estimate the class labels assigned to the input frames $\{{x_{t-M-1}, \ldots , x_t}\}$.

*Anticipation Head (Ant. Head)*: We use a linear layer (LN) and softmax to predict future phase probabilities $\{\hat{p_1}, \ldots , \hat{p_N}\}$ with shape $N \times C$.

Note that the regression task outputs $1 \times C$ values for the remaining time until the next phases, including the end-of-surgery (EOS) class. While remaining time regression could be performed via autoregressive decoding with a single iteration, we opt for a single-pass decoder since a single token can represent specific transitions over time and also due to the stronger performance on the classification task. Nevertheless, future work could explore generating multiple transitions per class (e.g. one for each generated token) to narrow potential performance gaps relative to an SP scheme.

SWAG-SP* (with Prior Knowledge Embedding). Our extracted prior knowledge captures the conditional probabilities of phase transitions at specific future times and is exclusively derived from the training set. Specifically, we compute the probability of having a future class *j* at time step $t+h_n\cdot 60$ given the current observed class *i*, denoted as $P(y_{t+h_n\cdot 60} = j \mid y_t = i)$. These probabilities form a tensor $P$, where each entry $P_{i,j,h_n\cdot 60}$ represents the likelihood of transitioning between classes at each anticipated time. We use these probabilities to initialise future token embeddings, assigning each future token at $t+h_n\cdot 60$ the probability vector $P_{i,:,h_n\cdot 60}$.

*SWAG-SP-R2C (regression to classification).* We demonstrate a possible mapping from regression outputs to classification via our regression-to-classification (R2C) method, which converts continuous time predictions into discrete phase intervals by sorting and binning predicted times. This approach can be used if one wishes to derive a phase sequence from a model originally trained for regression. Specifically, remaining time predictions for each class are mapped to a discrete sequence of *N* bins containing the predicted class integers $\hat{Y}_t=\{\hat{y}_0, \hat{y}_1, \ldots , \hat{y}_N\}$, in ascending order of occurrence. We use the current predicted class $\hat{y}_0$ to fill the sequence up to the first next class occurrence and use cross-entropy and mean squared error losses for the classification and regression tasks, respectively.

### Experimental setup

*Datasets*: We evaluate our work on two publicly available surgical phase datasets: *Cholec80* (C80) [32] and *AutoLaparo21* (AL21) [33]. For Cholec80, we adopt the same dataset splits as in [34], using 32 videos for training, 8 for validation, and 40 for testing. Similarly, for AL21, we apply the 10/4/7 split for training, validation, and testing, following [5, 33]. For the regression task, we follow a 60/20 train-test split as in [12, 13]. Both datasets were sampled at 1 frame per second (fps) following previous works [4, 20]. Across both datasets, we train on the 7 surgical phases, including an additional end-of-surgery (EOS) class, while disregarding tool annotations.

*Training details*: We train our model on a maximal horizon of $N=h_N$ minutes and evaluate it at intermediate steps without re-training. We use a simple weighted cross-entropy loss for the classification task and mean squared error loss for the regression task. Additional details on the training procedure are provided in the supplementary material (Sect. 1.1 in SM).

*Anticipation time*: Our method evaluates phase anticipation across multiple time horizons to assess both short-term and long-term predictive capabilities. This approach is justified by the substantial duration of procedures in our datasets: C80 training procedures average 38 min, while AL21 training videos average 66 min. By supervising our models on extended time horizons that encompass most of these workflows, we gain an important practical benefit: the ability to estimate the completion time of the entire surgical procedure from its earliest stages.

**Table 1:** Surgical phase recognition, anticipation, and segment-level performance

Methods	Cholec80				AutoLaparo21			
	Recognition		Anticipation		Recognition		Anticipation	
	Acc	F1	F1	SegF1	Acc	F1	F1	SegF1
Naive1$^\dagger$	**90**.**8**	91.1	29.3	24.5	70.7	71.0	19.6	16.4
Naive2$^\dagger$	90.8	**91**.**2**	**39**.**5**	11.9	69.4	69.3	34.3	10.7
AR	90.3	90.7	27.8	25.0	73.7	72.9	29.3	23.3
R2C	89.6	89.8	36.1	**32**.**5**	72.4	71.8	32.9	29.2
SP	88.3	88.8	29.4	23.8	**74**.**6**	**73**.**5**	38.4	30.8
SP$^*$	88.3	88.8	32.1	29.8	73.3	72.7	**41**.**3**	**34**.**8**

Frame-level metrics use weighted averaging to handle class imbalance. Recognition: accuracy/F1 score at current time ($t=0$). Anticipation: mean F1 score over horizon and IoU-based segment F1

Frame-level metrics use weighted averaging for class imbalance. Recognition: Acc/F1 at $t=0$. Anticipation: mean F1 over horizon. Segment: IoU-based F1 with EOS weighting (max 4/8 EOS samples per sequence). fBest performance highlighted, second-best underlined

$^\dagger$Baseline models (recognition only)

$^*$Prior knowledge initialisation

**Fig. 3:**
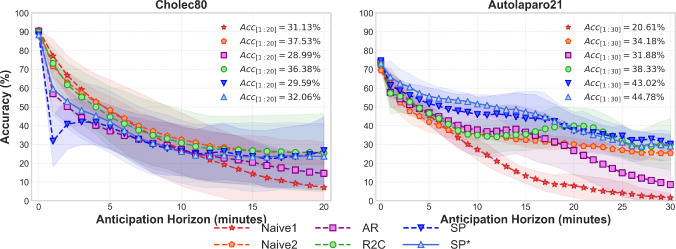
Models performance for surgical phase recognition and anticipation on Cholec80 and AutoLaparo21. Accuracy ($Acc_{[1:h_n]}$) over $h_n$ minutes (top right corners)

*Classification task*: Since long-term future phase classification has not been studied in previous work, direct comparison to other surgical workflow anticipation methods is not possible. Therefore, we assess our generative approaches against two baseline methods: a simple continuation model (Naive1) and a conditional-probabilistic model (Naive2). **Naive1** baseline extends the current recognised class over the future predicted horizon for *N* minutes. While effective for short-term predictions, its performance naturally degrades over time with new class transitions. **Naive2** baseline predicts future phases by sampling from the class probability distribution $P_{i,j,h_n\cdot 60}$, conditioned on the predicted current class $i$ and at the anticipated time index $h_n$. This baseline is effective due to the strong priors inherent in structured workflows, leveraging high recognition accuracy and precisely defined anticipation times.

**Fig. 4:**
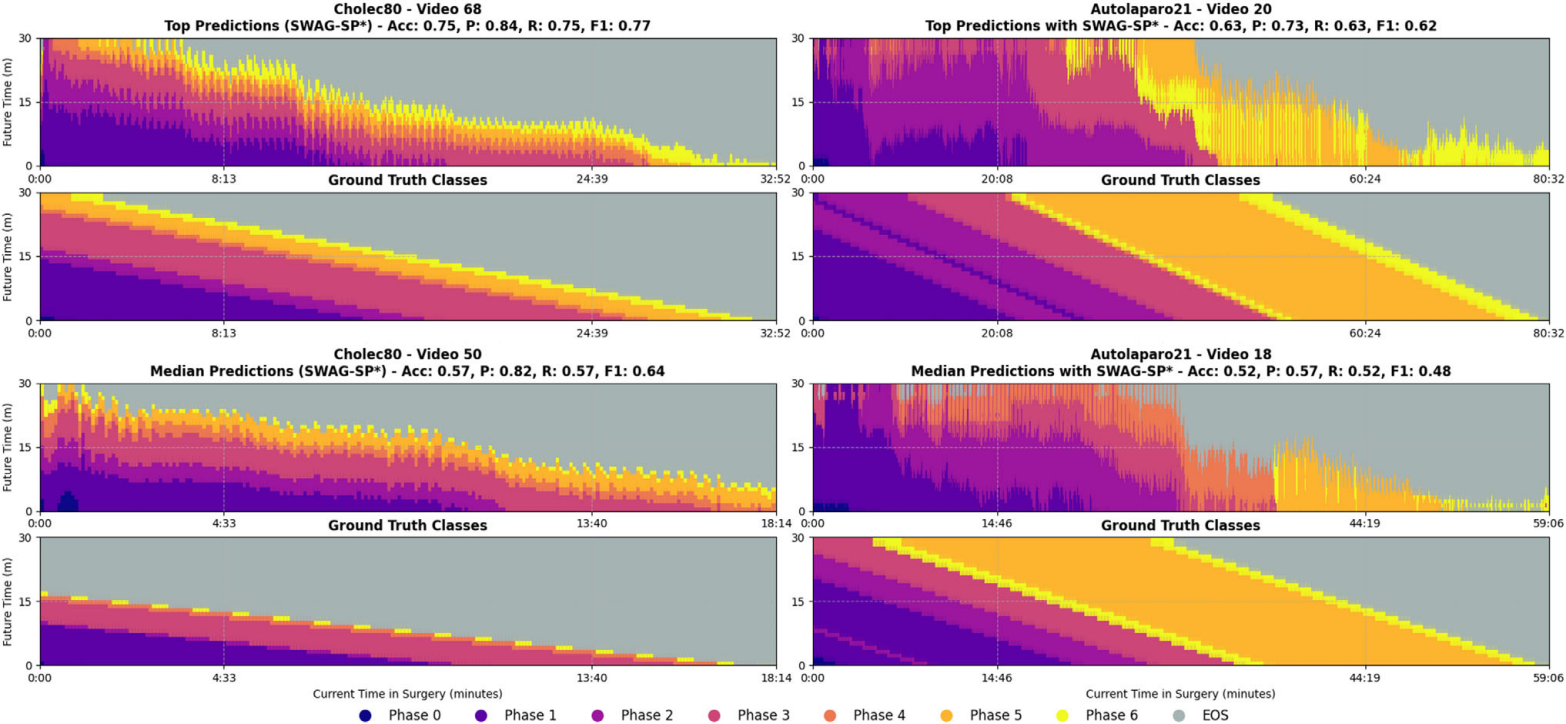
Qualitative results on high- and mid-performing test samples for phase recognition and anticipation using the SWAG-SP* model. Each block represents a single video from either Cholec80 or AutoLaparo21: The top two rows correspond to top-performing cases, and the bottom two to median-performing ones. For each video, the upper panel shows predicted phase segments over a 30-min anticipation horizon; the lower panel shows the corresponding ground truth. The *x*-axis indicates the current time within the surgery; the *y*-axis corresponds to the future anticipation horizon. Colours represent surgical phases, with grey denoting the end-of-surgery class

**Fig. 5:**
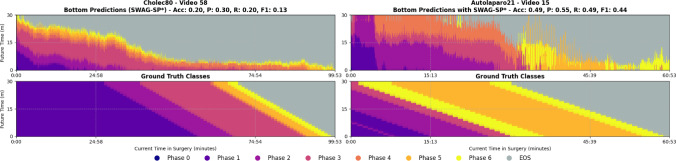
Qualitative results on two bottom-performing test samples using the SWAG-SP* model. Each example shows predicted (top) and ground-truth (bottom) phase segments over a 30-min anticipation horizon. The *x*-axis represents surgical time progression, and the *y*-axis denotes anticipation into the future. Colours indicate surgical phases; grey corresponds to the end-of-surgery class

*Regression task*: We include a secondary evaluation to compare our method to previous works. Specifically, we benchmark against Bayesian [12] and IIA-Net [13], both predicting the remaining time until the next-phase occurrence. IIA-Net relies on instrument presence and phase labels for supervision, while we solely use the latter. We did not include results from the Trans-SVNet [35] method as their evaluation was limited to a single horizon (5 min) and did not report the weighted MAE score (see Sect. Evaluation metrics). We extend the phase anticipation regression task to long horizons to include the remaining surgery duration (RSD) evaluation following previous works [7, 9, 10].

### Evaluation metrics

We use a combination of classification and regression metrics to evaluate our methodology. These metrics are calculated for each anticipation time, allowing us to analyse the model’s performance through time. For classification, we use weighted F1 scores which account for class imbalance by assigning importance proportional to class frequency. Surgical workflows are naturally imbalanced due to the inherent structure of procedural steps. To limit EOS class dominance, we cap EOS samples at 4 min for Cholec80 and 8 min for AutoLaparo21. We also introduce SegF1, a segment-based F1 score that evaluates temporal coherence by matching predicted segments to ground truth using IoU thresholding, penalising oversegmentation while rewarding correct phase boundaries. Detailed calculation is provided in the Supplementary Material. For regression tasks, we use the mean absolute error (MAE) to evaluate the model’s performance on predicting the remaining time until the next-phase occurrence. This metric quantifies the average absolute prediction errors in minutes. We use the *w*MAE, *in*MAE, and *out*MAE for *weighting* samples *outside* and *inside* the temporal horizon [12, 13]. Model selection is performed separately for each dataset using the validation set. For classification, we select the model with the highest overall mean weighted F1 score. For regression, we choose the model with the lowest weighted MAE.

## Results

### Surgical phase anticipation

Table 1 compares our models against two naive baselines. While Naive2 achieves strong anticipation performance on Cholec80 (F1 = 39.5%), our SP* model excels on the more challenging AutoLaparo21 dataset (F1 = 41.3%), demonstrating superior generalisation to complex, variable procedures. Notably, despite Naive2’s strong minute-level results, it performs poorly on multiple minutes intervals due to discontinuous predictions, whereas our approach maintains consistent segment-level (SegF1) performance. Figure 3 illustrates performance degradation across anticipation horizons.

Figures 4 and 5 provide qualitative comparisons for recognition and anticipation tasks across both datasets. Top-performing cases show predicted phase sequences that closely match ground truth timing and durations, while bottom-performing videos exhibit systematic temporal misalignment where predictions consistently over- or under-estimate phase lengths. The model maintains smooth, continuous predictions across all performance levels.

### Surgical phase anticipation with remaining time

Based on the classification evaluation results, we select the single-pass (SWAG-SP) decoder for comparison on state-of-the-art methods for remaining time until the next-phase occurrence (see Table 2). For 2-min and 3-min horizons, SWAG-SP achieves the best inMAE scores (0.54 and 0.77 min for 2 and 3 min), outperforming Bayesian [12] and IIA-Net [13]. At the 5-min horizon, SWAG-SP ranks second with its wMAE score, showing strong adaptability for mid-range anticipations.

**Table 2:** Remaining time to next-phase occurrence at 2, 3, and 5 min horizons

Methods	Cholec80 (60-20)								
	wMAE $[\downarrow ]$			inMAE $[\downarrow ]$			outMAE $[\downarrow ]$		
	2 min	3 min	5 min	2 min	3 min	5 min	2 min	3 min	5 min
Bayesian [12]	0.39	0.59	0.85	0.63	0.86	1.17	0.15	0.32	0.52
IIA-Net [13] $^\dag$	0.36	0.49	**0**.**68**	0.62	0.81	**1**.**08**	0.10	0.18	**0**.**28**
SWAG-SP	**0**.**32**	**0**.**48**	0.80	**0**.**54**	**0**.**77**	1.26	**0**.**09**	**0**.**17**	0.34

MAEs (in minutes) for *inside* and *outside* the anticipation window, and *weighted* mean. The results from previous methods are reported in IIA-Net [13]

The bold and underlined values are the best and second-best results, respectively

$^\dag$ Indicates methods using more data annotations for supervision i.e. bounding boxes and segmentation masks

IIA-Net [13] uses additional manual annotations like instrument bounding boxes and segmentation maps for supervision, while our approach relies solely on phase labels. In Table 3, we compare our SWAG-SP method with previous approaches on the remaining surgery duration (RSD) task. Using 4-fold cross-validation, our model ranks second on MAE-5 and MAE-ALL, outperforming previous methods, except BD-Net, all methods exclusively designed for this task.

**Table 3:** Remaining surgery duration (RSD) estimation on Cholec80

Methods	MAE-5 (min)	MAE-30 (min)	MAE-ALL (min)
TimeLSTM [6]	3.00 ± 1.87	5.30 ± 1.86	8.27 ± 6.25
RSDNet [7]	8.36 ± 3.71	6.83 ± 2.57	10.01 ± 6.54
CataNet [9]	2.47 ± 2.62	5.53 ± 2.75	8.27 ± 6.81
BD-Net [10]*	**1**.**97** ± 1.54	**4**.**84** ± **2**.**31**	**7**.**75** ± **6**.**43**
SWAG-SP (ours)	2.24 ± 2.32	6.48 ± 3.85	8.18 ± 5.33

Those results were reported in BD-Net

*This method randomly created 4-splits for validation, whereas we used consecutive 60/20 splits for training and testing, respectively

The bold and underlined values are the best and second-best results, respectively

**Fig. 6:**
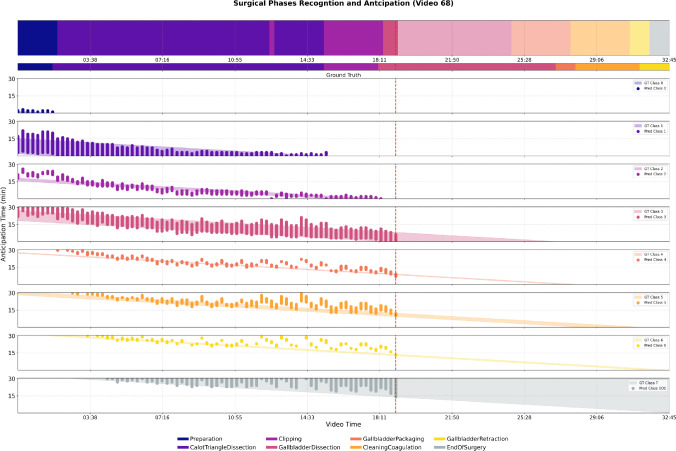
Surgical phase recognition and anticipation within a 30-min horizon (y-axis) for Cholec80’s video 68, with the current elapsed time (x-axis) indicated by the red dashed vertical line. The thin, second row shows the ground-truth phase segments, while the top rectangle displays the classified frames into phases; note that the more transparent colours to the right of the dashed line indicate the generated future phases at 60-s intervals. The last eight rectangles depict the previous remaining time predictions per class mapping to the 1-d timeline at the top

Figure 6 illustrates how predicted future phase segments would be depicted during inference. We extend the standard visualisation method for the recognition task by completing the missing part on the right side in an online setting with the predicted remaining surgical workflow. Our method proposed to fill this gap in a simple continuous approach using dense temporal segment classification, similarly to the recognition task but with a forward thinking approach. We believe this novel segment-based anticipation visualisation method could improve intraoperative awareness and guidance.

## Discussion

Our study reformulates surgical phase anticipation as a generative sequence modelling task, introducing SWAG with transformer-based approaches under autoregressive (AR) and single-pass (SP) decoding strategies, enhanced through token-level conditioning with prior knowledge (SP*).

Our results reveal distinct performance patterns that reflect the underlying complexity of different surgical procedures. On Cholec80, with its highly structured and predictable workflow, the Naive2 baseline achieves remarkably strong anticipation performance (F1 = 39.5%), demonstrating that simple approaches can excel when procedures follow consistent phase ordering. However, this advantage disappears on the more complex AutoLaparo21 dataset, where our SP* model demonstrates clear superiority (F1 = 41.3%, SegF1 = 34.8%), significantly outperforming naive baselines. This contrast highlights a fundamental insight: as surgical complexity increases, sophisticated modelling approaches become essential for accurate anticipation. The differential performance of our approaches provides valuable insights into anticipation strategies. Single-pass decoding with remaining time regression (R2C) achieves stronger performance on structured procedures like Cholec80 (F1 = 36.1% vs. 32.9% on AutoLaparo21), suggesting that regression-based approaches work well when phase durations are predictable. Conversely, sequence generation approaches (SP*) excel on variable procedures, better capturing the complex temporal dependencies and phase transitions characteristic of less standardised surgeries. The superior segment-level performance of our approaches compared to naive methods (despite similar frame-level scores) demonstrates the importance of maintaining temporal coherence in predictions. We successfully extend anticipation horizons beyond the previous 5-min limit, with SWAG-SP achieving the lowest wMAE and inMAE for short-term horizons while bridging into remaining surgery duration prediction. However, performance degrades significantly beyond 15–20 min horizons, with F1 scores dropping below 30% for most methods, indicating fundamental challenges in long-term surgical workflow prediction even for standardised procedures.

### Limitations and future directions

Our work reveals that long-term surgical anticipation remains challenging, with performance rapidly degrading over extended horizons due to the inherent unpredictability of intraoperative events and decision-making. Current limitations include reliance on relatively small datasets and the assumption of single valid future trajectories. Future work should prioritise three key directions: (1) incorporating uncertainty estimation to better handle the stochastic nature of surgical workflows, (2) developing evaluation frameworks that accommodate multiple plausible surgical trajectories rather than assuming deterministic workflows, and (3) scaling to larger, more diverse surgical datasets enriched with fine-grained action annotations and instrument control data. Looking towards longer-term advances, exploring generative approaches to create novel surgical scenarios beyond existing expert demonstrations could train robots to handle unprecedented cases, enhancing the current standard of surgical care and elevating patient safety to unprecedented levels.

## Conclusion

This work introduces SWAG, a unified encoder-decoder framework for surgical phase recognition and long-term anticipation, successfully demonstrating the value of generative sequence modelling enhanced with prior knowledge conditioning. Our evaluation across Cholec80 and AutoLaparo21 datasets reveals that while simple approaches suffice for structured procedures, sophisticated modelling becomes essential for complex, variable surgeries. SWAG’s superior performance on challenging datasets and its ability to maintain temporal coherence in predictions establishes it as a promising foundation for intraoperative guidance systems, while highlighting the fundamental challenges that remain in long-term surgical workflow anticipation.

## Supplementary Information

Below is the link to the electronic supplementary material.

Supplementary file 1 (pdf 4706 KB)
