# Supplementary material for: SWAG: long-term surgical workflow prediction with generative-based anticipation
Source: Int J Comput Assist Radiol Surg. 2025 Jun 26;20(11):2269–79. doi: 10.1007/s11548-025-03452-8 (PMC12575541; doi:10.1007/s11548-025-03452-8)
Supplement: Supplementary file 1 — (pdf 4706 KB) [file 11548_2025_3452_MOESM1_ESM.pdf]

1 Supplementary Material

1.1 Implementation Details

Our experiments were conducted on a single NVIDIA Tesla V100 GPU. We used a 12-head, 12-layer Transformer encoder as our spatial feature extractor, based on the ViT-B/16 architecture following LoViT. This model was pre-trained on ImageNet 1K (IN1k) and produced 768-d representations, with an input image size of 248×248 pixels. For training the spatial feature extractor, we used stochastic gradient descent with momentum for 35 epochs, with a 5-epoch warm-up period and a 30-epoch cosine annealed decay. We used a batch size of 16 and a learning rate of 0.1, which was multiplied by 0.1 at the 20th and 30th epochs. We set the weight decay to 1e-4 and the momentum to 0.9. For the Temporal Self-Attention, we used an input clip length l of 1440 frames or 24 minutes at 1fps with a sliding context length window w of 20 frames, generating 512-d feature vectors. Key-pooled feature dimensions d are 64-d and 32-d on Cholec80 and AutoLaparo21, respectively. The temporal modules underwent training for 40 epochs using SGD and momentum with a learning rate of 3e-4, weight decay of 1e-5, a 5 epoch warm-up period, and a 35 epoch cosine annealed decay, with a batch size of 8.

1.2 Future Tokens Embedding Initialization

This process involves iterating over all future token indices h_n to generate the probability vector $\mathbf{p}_t$. We sample from the transition probability matrix $\mathbf{P}$ using the current class index i, which corresponds to the ground-truth label when using teacher forcing during training, or the model's predicted class otherwise. The index h_n represents the position of the future token within the anticipation horizon h_N. We define the transition probability of being in a future class j given the current class i as follows:

$$p(y_{t+h_n \cdot 60} = j \mid y_t = i) = \mathbf{P}[i, j, h_n] \tag{1}$$

with

$$i \in \{0, \ldots, C-1\}, \quad j \in \{0, \ldots, C\}, \quad h_n \in \{h_1, \ldots, h_N\} \tag{2}$$

where C represents the total number of surgical phases, and C corresponds to the additional end-of-sequence (EOS) class included for padding purposes. We define the probability vector $\mathbf{p}_t$ as a collection of class probability vectors for each future horizon h_n:

$$\mathbf{p}_t = \big[\mathbf{P}[i, :, h_n]\big]_{n=1}^{N}, \quad h_n \in \{h_1, \ldots, h_N\} \tag{3}$$

where $\mathbf{P}[i, :, h_n]$ corresponds to the probability vector for the future token at index h_n, sampled from the i-th row of the matrix $\mathbf{P}$, which is based on the current class index. Each future token embedding $\mathbf{q}_t$ is computed by combining an embedding u_t, initialized using the Xavier uniform distribution, with the linearly transformed higher-dimensional probability vector $\mathbf{p'}_t$ and a sinusoidal positional encoding. The final embedding serves as the input to the transformer decoder:

$$\mathbf{p'}_t = W_p \mathbf{p}_t + \text{bias}_p \tag{4}$$

$$\mathbf{q}_t = \text{LayerNorm}(u_t + \alpha \mathbf{p'}_t + \text{PositionalEncoding}(t)) \tag{5}$$

1.3 Additional Results

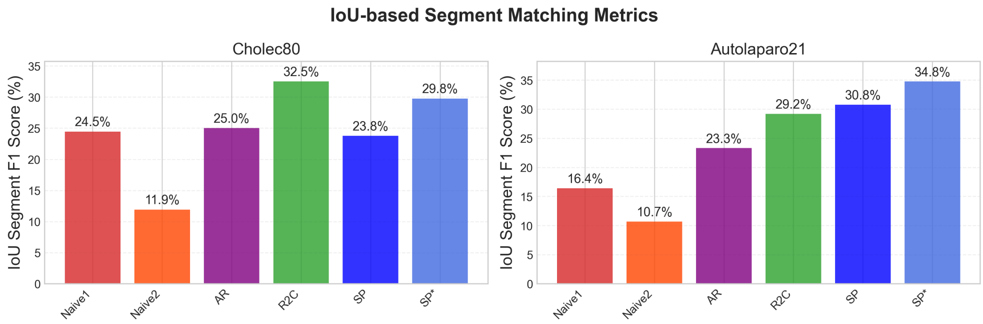

Fig. 1: Methods IoU performance for surgical phase recognition and anticipation on Cholec80 and AutoLaparo21.

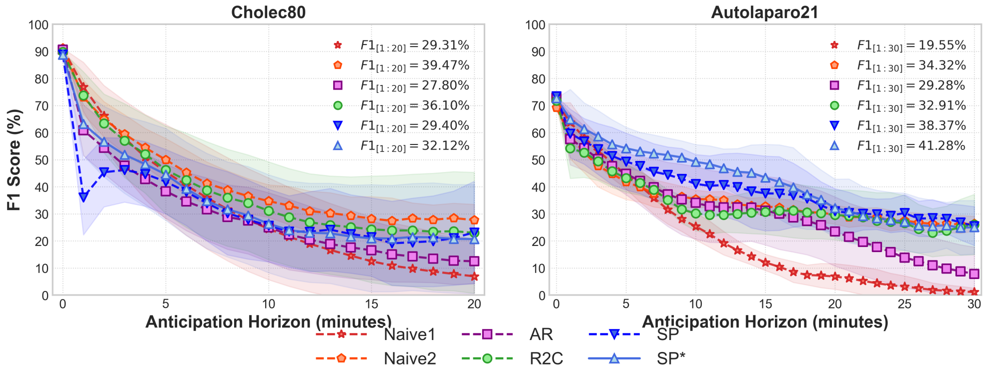

Fig. 2: Methods performance for surgical phase recognition and anticipation on Cholec80 and AutoLaparo21. We report the frame-level accuracy scores $Acc_{[1:h_n]}$ and show the mean anticipation scores over h_n minutes (top right corners).

2 Segment-based F1 Score (SegF1) Calculation

The SegF1 metric evaluates temporal coherence in surgical phase predictions by matching continuous segments rather than individual frames, addressing the problem of oversegmentation.

2.1 Methodology

Given a current time point t, our model anticipates future class labels at 1-minute intervals across a fixed anticipation horizon of N minutes. Let:

- $\hat{y}_{t+h_n \cdot 60}$ denote the predicted label at minute index $h_n \in \{1, \ldots, N\}$
- $y_{t+h_n \cdot 60}$ denote the corresponding ground-truth label

We define the sequences over the horizon as:

$$\hat{\mathbf{y}}_{\mathbf{t}} = (\hat{y}_{t+60}, \hat{y}_{t+2\cdot 60}, \ldots, \hat{y}_{t+N\cdot 60}), \quad \mathbf{y}_{\mathbf{t}} = (y_{t+60}, y_{t+2\cdot 60}, \ldots, y_{t+N\cdot 60})$$

2.1.1 Segment Identification

From these sequences, we identify continuous segments of the same class. A segment $S = (start, end, c)$ represents consecutive minutes with the same class label c, where $start$ and end are minute indices.

The set of ground truth segments $\mathcal{S}^{GT}$ is derived from $\mathbf{y}_{\mathbf{t}}$, and the set of predicted segments $\mathcal{S}^{PR}$ is derived from $\hat{\mathbf{y}}_{\mathbf{t}}$.

2.1.2 Intersection over Union (IoU) Calculation

For two segments $S_i = (start_i, end_i, c_i)$ and $S_j = (start_j, end_j, c_j)$, the Intersection over Union (IoU) is:

$$\mathrm{IoU}(S_i, S_j) = \frac{|\mathrm{Intersection}(S_i, S_j)|}{|\mathrm{Union}(S_i, S_j)|} \tag{6}$$

$$= \frac{\max(0, \min(end_i, end_j) - \max(start_i, start_j) + 1)}{(end_i - start_i + 1) + (end_j - start_j + 1) - |\mathrm{Intersection}(S_i, S_j)|} \tag{7}$$

2.1.3 Optimal Matching with Hungarian Algorithm

A valid match between predicted segment $\hat{S}_i \in \mathcal{S}^{PR}$ and ground truth segment $S_j \in \mathcal{S}^{GT}$ requires:

$$\hat{c}_i = c_j \quad \text{and} \quad \mathrm{IoU}(\hat{S}_i, S_j) \geq \tau$$

where $\tau = 0.25$ in our implementation, requiring at least 25% overlap.

We construct a cost matrix C where $C_{i,j} = -\mathrm{IoU}(\hat{S}_i, S_j)$ if $\hat{c}_i = c_j$, otherwise $C_{i,j} = \infty$. Using the Hungarian algorithm, we find the optimal matching $\mathcal{M}$ between predicted and ground truth segments.

2.1.4 Metric Calculation

Based on the matching results:

$$\mathrm{TP} = |\mathcal{M}|, \quad \mathrm{FP} = |\mathcal{S}^{PR}| - |\mathcal{M}|, \quad \mathrm{FN} = |\mathcal{S}^{GT}| - |\mathcal{M}|$$

The SegF1 is then calculated as:

$$\text{Precision} = \frac{\text{TP}}{\text{TP} + \text{FP}} \tag{8}$$

$$\text{Recall} = \frac{\text{TP}}{\text{TP} + \text{FN}} \tag{9}$$

$$\text{SegF1} = \frac{2 \cdot \text{Precision} \cdot \text{Recall}}{\text{Precision} + \text{Recall}} \tag{10}$$

2.2 EOS Class Handling

For the End-of-Surgery (EOS) class, we apply special handling to prevent its dominance:

- We limit the number of EOS frames considered per sequence to $\ell_{EoS} = 4$ minutes for Cholec80 and $\ell_{EoS} = 8$ minutes for AutoLaparo21
- We apply a weighting factor of 0.5 to EOS segments in the calculation of TP, FP, and FN

Implementation parameters:

- IoU threshold (τ): 0.25
- EOS class weighting: 0.5
- End-of-Surgery consideration length (ℓ_{EoS}): 4 minutes (Cholec80), 8 minutes (AutoLaparo21)
- Anticipation horizon: 20 minutes (Cholec80), 30 minutes (AutoLaparo21)

2.3 Ablation Studies

We conducted ablation experiments to study the impact of various factors on model performance, including context length, anticipation interval, number of context tokens, temporal pooling methods, and model size.

Context Length. Figure 3 shows that a context length of 24 minutes yields the highest mean cumulative accuracy on both datasets.

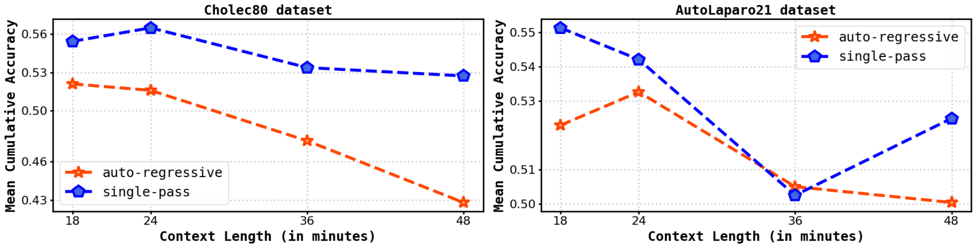

Fig. 3: Context Length (in minutes) of the input sequence.

Compression and Anticipation Time. Figure 4 indicates that a 1-minute interval between input samples produces the highest accuracies on both datasets.

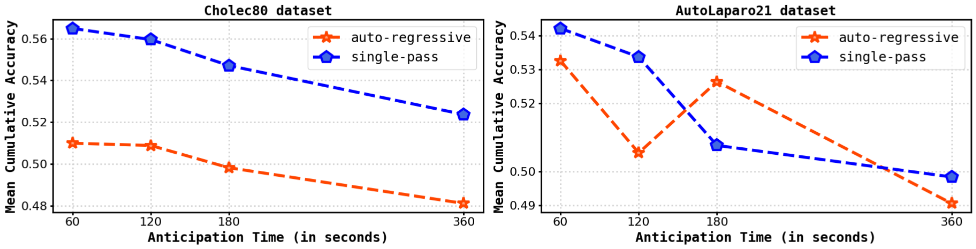

Fig. 4: Effect of the anticipation time between frames during training (in seconds) on the test accuracy.

Temporal Pooling Methods. Figure 5 compares global and interval pooling methods. Single-pass decoding with global context tokens consistently outperforms auto-regressive decoding with either temporal pooling methods for all numbers of context tokens and on both datasets.

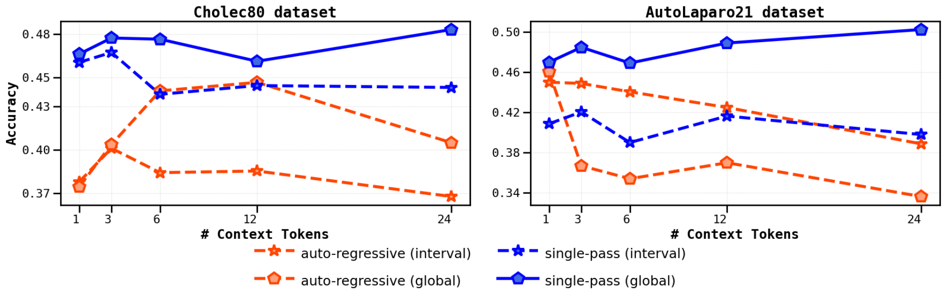

Fig. 5: Number of context tokens with a fixed context length of 24 minutes i.e. compression of input sequence with global and interval temporal pooling methods.

Model Size. Figure 6 demonstrates that medium-sized models achieve the best overall accuracies, with the optimal model size varying slightly between datasets. These ablations indicate that careful tuning of these parameters can significantly impact long-term surgical phase anticipation performance.

Inside horizon MAE per class. On the Cholec80 dataset, we observe an increasing error from approximately 2 to 8 minutes for mid- to late-stage surgical phases, specifically classes 4, 5, 6, and EOS. In contrast, anticipation errors for phases 2 and 3 remain stable over time, averaging around 5.5 minutes. The model exhibits an error below 1 minute for phase 1, likely because phase 1 occurs at the very beginning of the surgery, leading the model to predict low values that are often accurate.

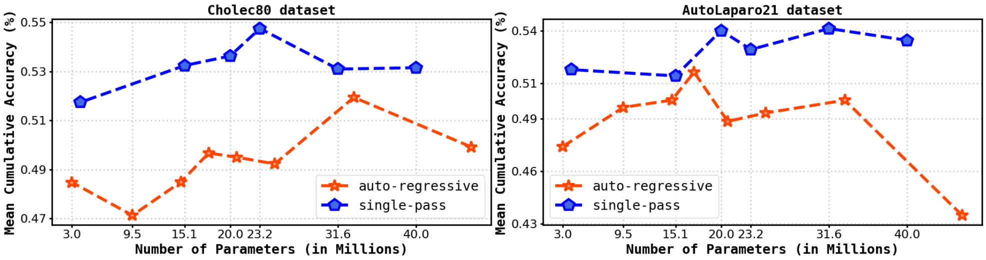

Fig. 6: Model size (million parameters) over accuracy.

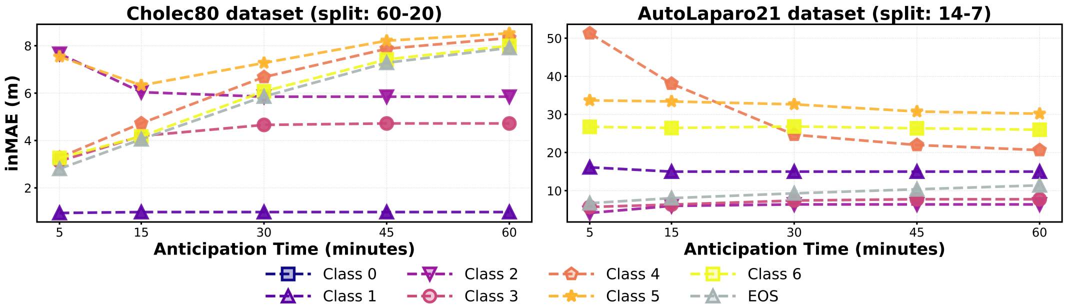

Fig. 7: MAEs inside the anticipation horizons per-class on Cholec80 and AutoLaparo21.

For the AutoLaparo21 dataset, the task is more challenging, especially for mid- to late-phase stages, though the EOS class is less impacted. Errors are less correlated with time, reflecting the intrinsic uncertainty of phase variability. Classes 4, 5, and 6, in particular, are inconsistently present and often exhibit abrupt transitions, making prediction difficult. Over long horizons, the model takes a conservative approach by predicting mid-range values. However, as it nears the 5-minute horizon, it attempts to predict lower, more specific values, which can lead to substantial errors if the phase is absent or has sudden transitions.

2.4 Our SWAG Application

We show how a pre-trained language model can translate the predictions into language. Available on our project homepage: https://maxboels.github.io/swag

2.5 Additional Qualitative Results

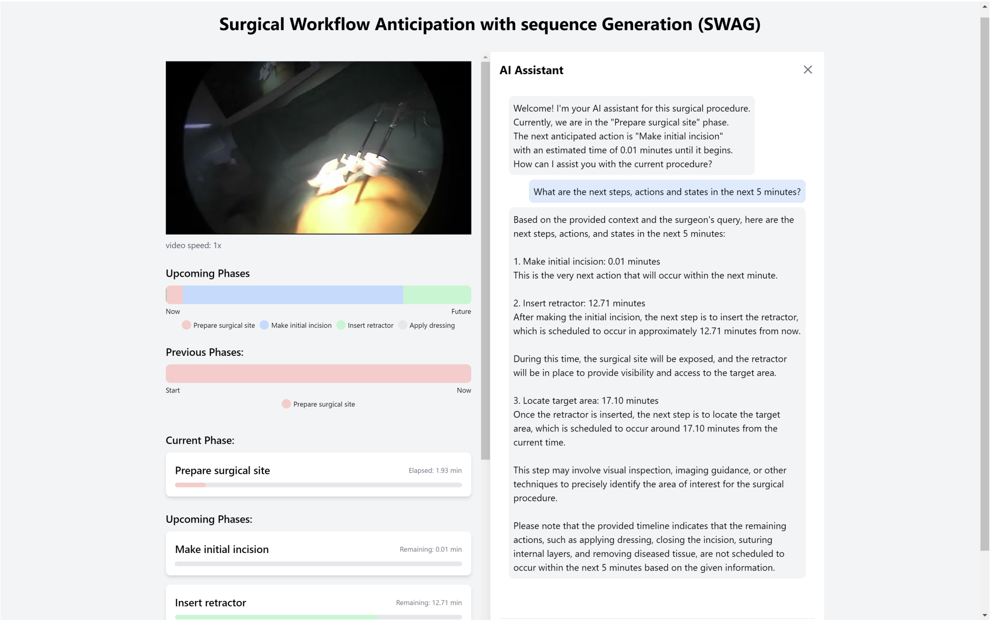

Fig. 8: SWAG + LLM application.

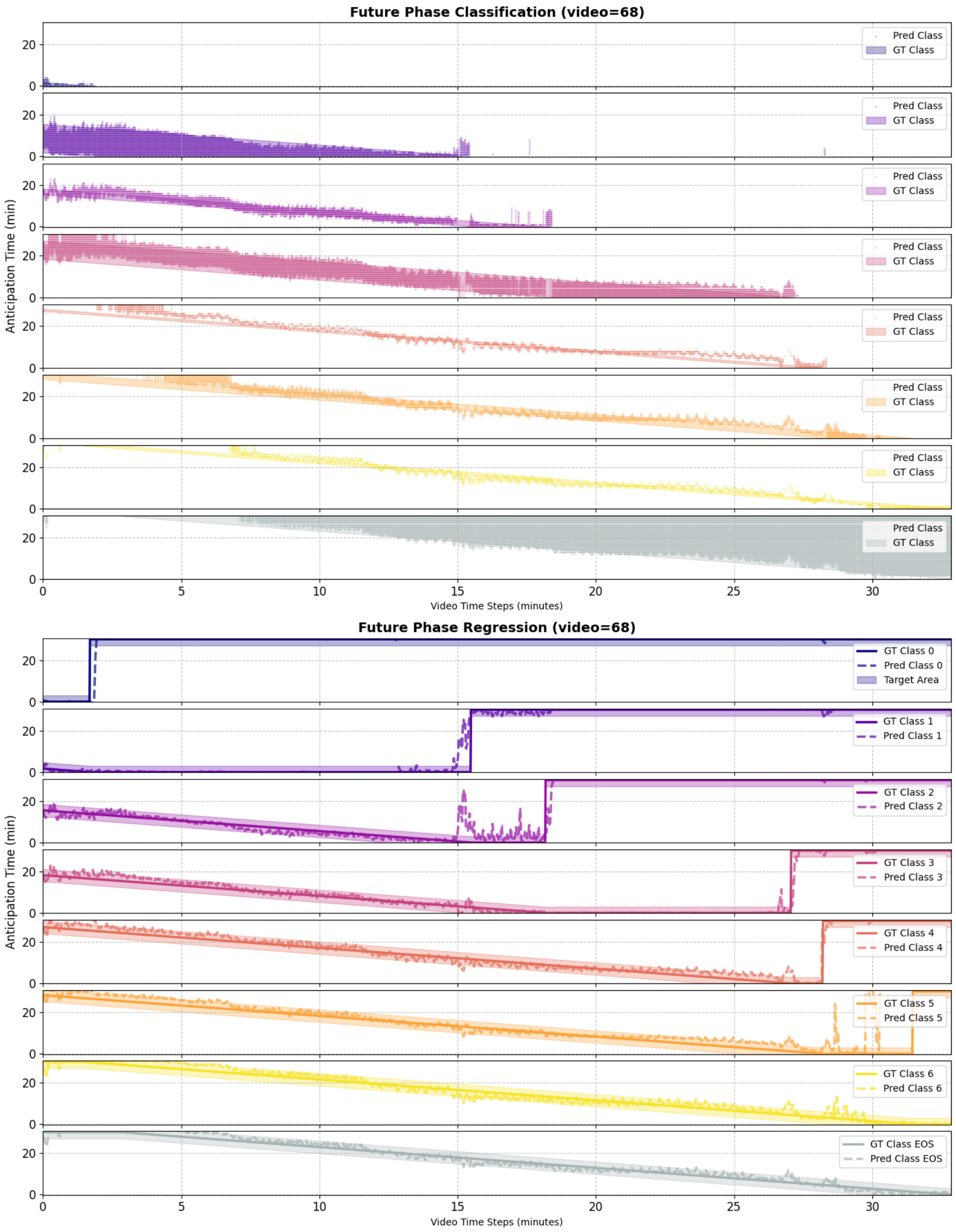

Fig. 9: Comparison of future phases classification (top) and regression (bottom) task on video 68 from the cholec80 dataset.
